# A rare case of metastatic myxofibrosarcoma to the right colon

**DOI:** 10.1093/jscr/rjaf635

**Published:** 2025-10-06

**Authors:** Timothy K Farrell, Matthew A Carnell, Vincent J Obias

**Affiliations:** Colorectal Research Unit, Department of Surgery, Johns Hopkins School of Medicine, 1800 Orleans St, Baltimore, MD 21287, United States; Department of Surgery, Walter Reed National Military Medical Center, 8901 Wisconsin Ave, Bethesda, MD 20889, United States; Colorectal Research Unit, Department of Surgery, Johns Hopkins School of Medicine, 1800 Orleans St, Baltimore, MD 21287, United States; Department of Surgery, Macon & Joan Brock Virginia Health Sciences at Old Dominion University, 5115 Hampton Blvd, Norfolk, VA 23529, United States; Colorectal Research Unit, Department of Surgery, Johns Hopkins School of Medicine, 1800 Orleans St, Baltimore, MD 21287, United States

**Keywords:** myxofibrosarcoma, colectomy, case report, soft tissue sarcoma, cecal mass, metastasis

## Abstract

Myxofibrosarcoma (MFS) is a common soft tissue sarcoma typically arising in the extremities of older adults. Metastatic spread usually involves the lungs, lymph nodes, or bone. We describe a rare case of metastatic MFS to the colon in a 76-year-old man, three years after surgical excision and radiation treatment for a left flank primary tumor. The patient presented with symptoms of obstipation and was found to have a 4 cm cecal mass confirmed by imaging and colonoscopy. Biopsy and surgical pathology confirmed metastatic MFS. A right colectomy was performed without complication. This case highlights an uncommon site of MFS metastasis and underscores the importance of long-term surveillance in high-grade tumors, including evaluation of the gastrointestinal tract when symptoms arise.

## Introduction

Myxofibrosarcoma (MFS) is a connective tissue malignancy which typically presents in the extremities of adults in the sixth to eighth decade of life [1]. These tumors arise as painless, slow-growing masses and recur in 7%–15% of treated cases and metastasize in 20%–35% of cases [2, 3]. Workup of these tumor diagnoses typically includes a thorough history, physical examination, and imaging, including magnetic resonance imaging (MRI) and computed tomography (CT). MRI provides the best diagnostic information for identifying the extent of tumor infiltration.

MFS tumors are more likely to recur locally than to metastasize. Therefore, the mainstay treatment option remains surgical resection with neoadjuvant or adjuvant radiotherapy (RT) depending on the degree of tumor infiltration revealed in preoperative imaging and identified along surgical margins during pathologic staging. For cases in which the tumor recurs or metastasizes, the primary treatment option also remains wide local excision. This can result in patients with MFS undergoing multiple procedures [4]. In this case report, we document what we believe is the second known case of metastatic MFS to the right colon, an extremely atypical site for MF metastatic disease occurrence.

## Case report

The patient is a 76-year-old male diagnosed with a left flank MFS in 2021; his past medical history includes hypertension and unprovoked deep vein thrombus, anticoagulation therapy, and a diagnosis of prostate cancer, which is in remission having been treated with brachytherapy. Upon admission, the patient reported a left flank mass that had been growing over a period of several months. This was evaluated using ultrasound (US) and MRI imaging, which identified a mass measuring 8.7 × 4.9 × 4.9 cm, suggestive of a MFS. Subsequently, a core needle biopsy confirmed the diagnosis of MFS. A wide local tissue excision was performed without complication. The final pathology assessment of the excised tissue indicated a high-grade, 8.8 cm MFS tumor. The final stage of the tumor was pT2N0M0G3. The patient received adjuvant RT because the deep surgical margin was < 2 mm, indicating a close resection margin on the deep surface of the surgical specimen. Unfortunately, the patient’s subsequent medical surveillance plan is not well documented in his electronic health record.

**Figure 1:**
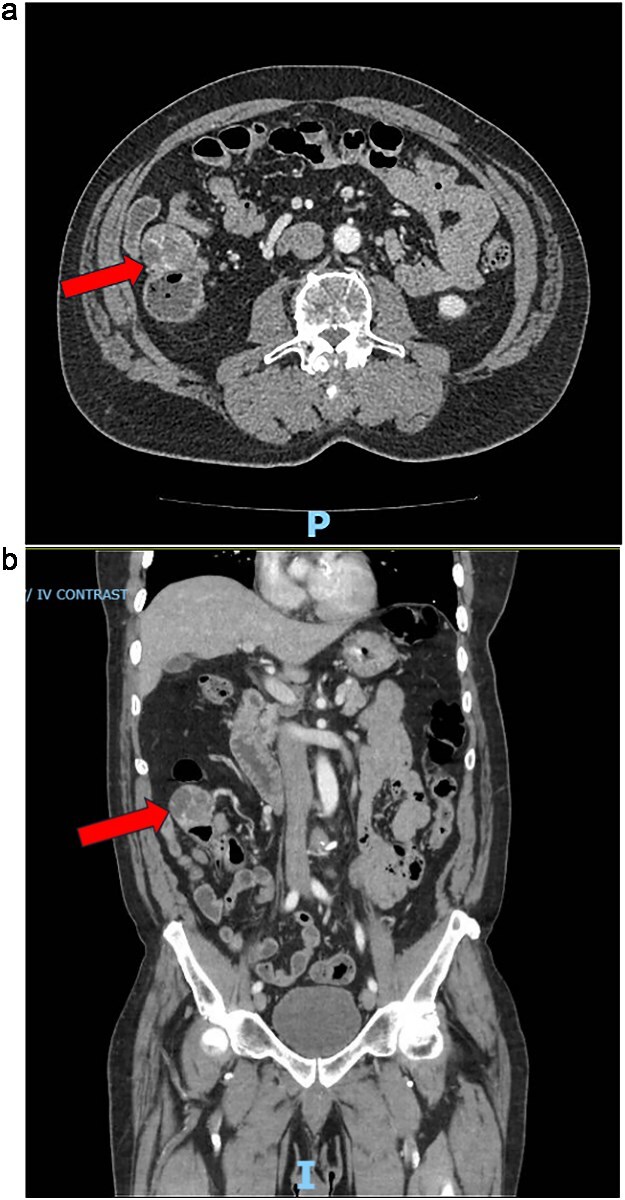
(a) CT abdomen/pelvis axial and coronal views during emergency department presentation with cecal mass seen. (b) CT abdomen/pelvis axial and coronal views during emergency department presentation with cecal mass seen.

**Figure 2:**
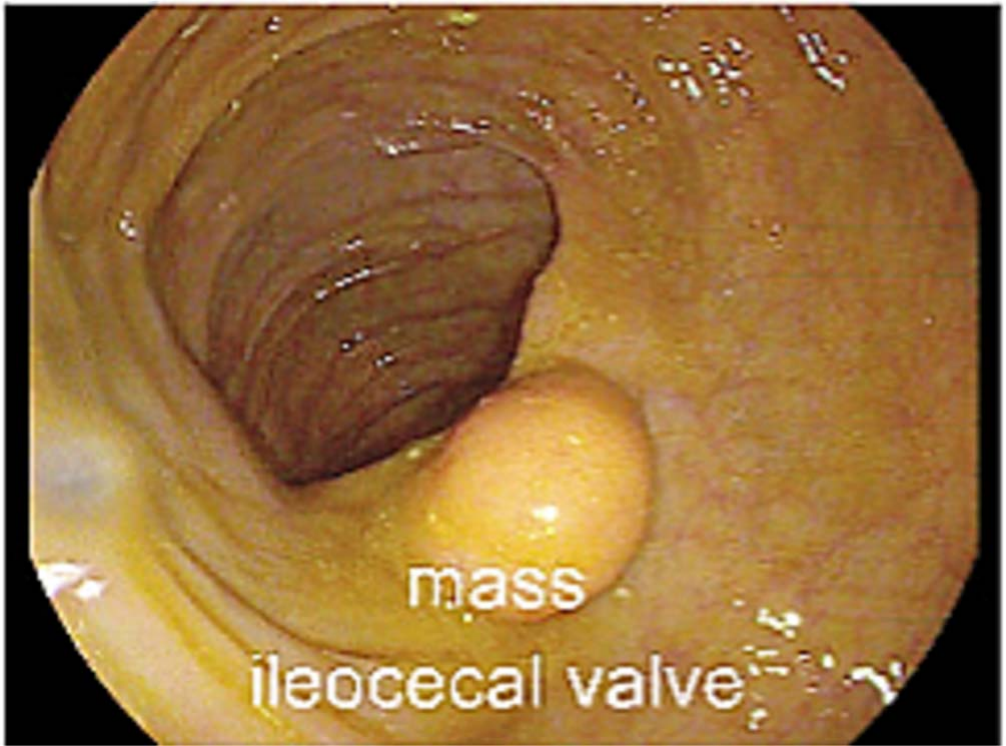
Cecal mass seen during colonoscopic evaluation.

Two years following his MFS diagnosis and treatment, the patient developed new symptoms. After sustaining a fall, the patient complained of persistent weakness, nausea, and anorexia. At the time, the patient was hemodynamically normal and had a normal laboratory evaluation. However, these assessments noted the presence of a 4 cm cecal mass on a CT scan of the abdomen and pelvis (Fig. 1a and b). The patient was admitted for generalized weakness and expedited workup of the cecal mass, which was biopsied and confirmed to be a metastatic MFS. Next-Gen Sequencing completed on this specimen was negative for Cancer Hotspot mutations. A colonoscopy was performed, which showed no other synchronous colonic lesions. However, the colonoscopy identified a submucosal mass located at the ileocecal valve (Fig. 2).

**Figure 3:**
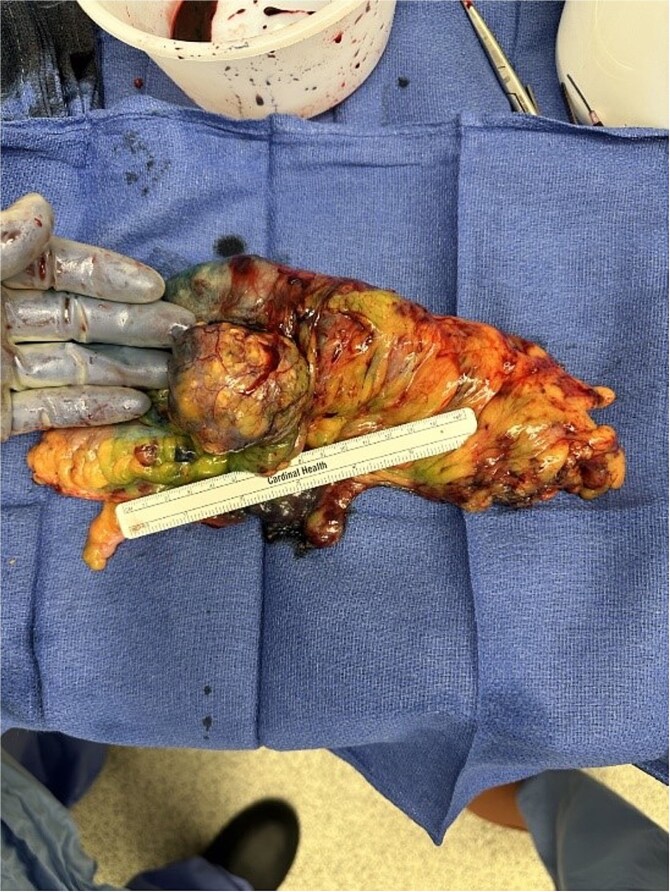
Ex-vivo gross surgical specimen following right colectomy.

After a discussion with a multidisciplinary tumor board, the decision was made to proceed with primary resection of the mass. The patient underwent an uncomplicated right colectomy. The ex-vivo specimen can be seen in Fig. 3. He tolerated the procedure well and recovered appropriately. He was discharged to a subacute rehab facility on postoperative Day 6. After a short stay in the rehab facility, the patient was seen in follow-up and was recovering well from surgery. The final pathology report on his surgical specimen confirmed a metastatic MFS, high-grade, forming a 5.5 cm mass with negative surgical margins. There was no lymph node involvement. The patient is following up with medical and radiation oncology.

## Discussion

The median time for recurrence of MFS is 21 months. Even though this patient underwent post-operative radiation, current data demonstrates that radiation does not affect the metastatic potential of the tumor [5]. The metastatic potential of soft tissue sarcomas is more dependent on the pathologic grade of the tumor, which was high for this patient’s primary tumor. High-grade MFS have a metastasis rate of 18%–47% [6]. Other risk factors associated with increased metastatic potential in soft tissue sarcomas treated with radiation include a tumor size > 5 cm; location in the head, neck, or deep trunk; and a patient age > 64 years [7]. This patient had all these concerning features (tumor size, location, and patient age). However, the colon is an extremely atypical location despite this patient’s elevated risk for metastasis.

MFS is a common soft tissue tumor. Given this patient’s high-grade primary tumor, surveillance is crucial to favorable outcomes and quickly identifying metastasis. This appears to be only the second colonic metastasis of MFS documented in the literature. While the colon is a rare location for an MFS, it is crucial for diagnosticians to be aware of this potential in providing proper patient care. Special attention should be given when patients with a history of MFS develop new gastrointestinal symptoms. Further studies should be completed to elucidate which factors influence atypical spread of MFS.

## Conclusion

Metastatic MFS in the colon is exceedingly rare, with only two documented cases including this report. Clinicians should maintain a high index of suspicion for atypical metastatic spread in patients with high-grade MFS, particularly when gastrointestinal symptoms develop. Comprehensive and prolonged surveillance is warranted in such cases to ensure early detection and management of metastasis.
